# Electromechanical–Thermal Coupling Modeling of Scattering Fields for Conformal Load-Bearing Antennas

**DOI:** 10.3390/mi17091098

**Published:** 2026-09-18

**Authors:** Yan Wang, Peiyan Zhang, Jiayang Li, Linchen Han, Longyang Wang, Peiyuan Lian, Zhihai Wang, Wanlu Hu, Congsi Wang

**Affiliations:** 1 College of Computer and Information Engineering, Xi’an University of Architecture and Technology, Xi’an 710055, China; wangyan5169@163.com (Y.W.); 17730615675@163.com (J.L.); hanlinchen2002@163.com (L.H.); 2 College of Artificial Intelligence and Robotics, Xi’an University of Architecture and Technology, Xi’an 710055, China; 3 State Key Laboratory of Electromechanical Integrated Manufacturing of High-Performance Electronic Equipments, Xidian University, Xi’an 710071, China; wangly268@163.com (L.W.); lian100fen@126.com (P.L.); 4 CETC No. 38 Research Institute, Hefei 230088, China; z11040925@163.com (Z.W.); tmhwl1995@163.com (W.H.); 5 Guangzhou Institute of Technology, Xidian University, Guangzhou 510555, China

**Keywords:** conformal load-bearing antennas, electromechanical–thermal coupling, scattering field modeling, radar cross section, multi-physics coupling, aerodynamic loads

## Abstract

During service, conformal load-bearing antennas (CLBAs) are subjected to the coupled effects of aerodynamic and aerothermal loads. The resulting geometric distortion of the array surface, deflection of element pointing, and temperature drift of the material electromagnetic parameters lead to the degradation of radar cross-section (RCS) characteristics. To overcome the limitation of existing scattering models in uniformly describing the aforementioned multi-physics coupling effects, this paper proposes a comprehensive electromechanical–thermal coupled modeling method for the scattering field of CLBAs. This method establishes a complete mapping from flight conditions to the array RCS by incorporating geometric corrections for element-level pointing deflection and bending deformation, material corrections accounting for the temperature-dependent antenna efficiency, and phase corrections induced by aerodynamic displacements. Verification using a 9 × 9 cylindrical conformal array shows that, within a scanning range of ±30°, the model calculations agree with HFSS full-wave simulations with an absolute error of less than 1 dB, and the broadside RCS is reduced by 14.97 dB compared with that of a planar array. Furthermore, a BP neural network surrogate model is constructed to achieve accurate prediction of the array physical fields. Analyses across the Mach regime of 0.20–0.65 Ma indicate that structural deformation is the dominant cause of RCS distortion, with the trailing-edge array experiencing a rapid nonlinear increase in RCS peak increment, reaching up to 7 dB at 0.65 Ma. The proposed model provides an effective theoretical tool for the rapid evaluation of stealth performance for conformal antennas operating in complex environments.

## 1. Introduction

Conformal load-bearing antennas (CLBAs) integrate radio-frequency functionality with load-bearing structures, representing a key enabling technology for the integrated “structure–function–stealth” design of next-generation flight vehicles [1,2,3]. In service, however, the wing skin is subjected to long-term coupled aerodynamic and aerothermal loads [4,5,6], which induce multi-physics effects involving fluid–structure–thermal interactions. These effects lead to geometric distortion of the array surface, displacement of array elements, and temperature-dependent drift of material electromagnetic parameters, further exacerbating the inherent issues of non-uniform element arrangement and beam-pointing deflection caused by the curved substrate of conformal carriers [7,8,9,10]. Consequently, the scattering performance of the conformal load-bearing antenna is also degraded.

Antenna scattering typically comprises two components: the structural-mode scattering field, which is determined by the physical structure of the antenna, and the antenna-mode scattering, which is directly associated with antenna matching and radiation performance [11]. In terms of fundamental antenna scattering theory, Munk B. systematically summarized the theoretical framework for antenna scattering analysis, explicitly indicating that the structural-mode scattering field should be defined as the residual component after subtracting the antenna-mode scattering field (induced by reradiation) from the total scattering field, and its magnitude is not explicitly dependent on the complexity of the antenna physical structure [12]. Steyskal H., based on an equivalent-circuit analytical model for antenna scattering and reception states, investigated the relationship between the scattered and absorbed energy amplitudes and explored the fundamental limits of antenna scattering fields [13]. The above-mentioned studies have laid the theoretical foundation for antenna scattering characteristic analysis; however, they all focus on isolated antenna elements or ideal planar arrays and do not address the influence of structural deformation on scattering fields.

Regarding the influence of structural deformation on array antenna performance, Ossowska et al. simulated the effects of three structural deformation patterns—random, symmetric, and asymmetric—on the performance of high-resolution wide-swath synthetic aperture radar (SAR) systems, explicitly demonstrating that structural deformation directly leads to performance degradation in the elevation direction. However, their analysis was confined to specific SAR application scenarios and did not establish a general analytical relationship between deformation parameters and electrical performance [14]. Zaitsev and Hoffman quantitatively investigated the radiation characteristics of phased array antennas under various typical deformation patterns, revealing the correlation between planar root-mean-square (RMS) errors and gain degradation, yet they did not address the corresponding scattering characteristics [15]. Arnold analyzed the bending deformation of wing-mounted phased array antennas induced by aerodynamic loads during flight, indicating that structural deformation results in phase center errors, elevated sidelobe levels, and beam-pointing deviations. Nevertheless, this study remained at a qualitative level and did not establish a quantitative mapping model between the element-level displacement field and the scattering field [16]. Famoriji et al. proposed a structural–electromagnetic coupling statistical model for millimeter-wave antenna arrays, quantitatively analyzing performance variations under random position errors and saddle-shaped distortions. However, this model relies on finite-element numerical simulations as an intermediate step and has not yet achieved an analytical expression from load conditions to electrical performance responses, making it difficult to support rapid parametric evaluation [17]. The above studies have revealed, from various perspectives, the influence of structural deformation on array antenna performance. Nevertheless, the research objects are predominantly planar arrays, and most studies focus on radiation performance, with comparatively insufficient attention given to scattering performance.

To address the challenges of large curvature and RCS reduction in wing-leading-edge conformal arrays, Xu et al. proposed a dual-polarized ultra-wideband leading-edge conformal tightly coupled dipole array. Based on the scattering matrix method, they achieved rapid conversion from planar elements to conformal elements and explicitly noted that most existing studies focus on radiation characteristics, leaving a gap in scattering characteristic research [18]. Lian et al., based on the infinitesimal dipole model, proposed a method for analyzing the scattering characteristics of cylindrical conformal arrays with variable curvature, employing osculating circles to fit the variable-curvature array structure while accounting for the effects of structural deformation and mutual coupling among elements. However, the deformation in this method originates from prescribed geometric curvature variations rather than being driven by the physical mechanisms of aerodynamic loads, and the simultaneous effects of temperature fields on material electromagnetic parameters are not considered [19].

In terms of scattering modeling and performance analysis methods, Zhang et al. established an equivalent network model for phased array antennas. By incorporating independent matching networks behind each element and extracting network parameters under different terminated loads, they achieved co-design of scattering reduction and radiation performance. This study provides an effective circuit-level approach for regulating the scattering performance of array antennas; however, the modeling target is an ideal planar array, and the inherent geometric distortion of conformal carriers is not taken into account [20]. Li et al. also adopted the equivalent network model, investigating the intrinsic relationship between element radiation fields and array scattering fields, and estimating the antenna-mode scattering field from simulated radiation field data, thereby establishing a conversion pathway from radiation performance to scattering performance. Nevertheless, this approach relies on radiation field simulation data as input, while the radiation field itself undergoes alterations owing to structural deformation and thermal effects under service conditions, and thus fails to establish a direct mapping from external loads to scattering fields [21]. Wang et al. developed a statistical model for scattering performance incorporating structural deformation and random position errors, and reformulated the multi-objective optimization problem of comprehensive radiation and scattering compensation into a single-objective problem by constructing a fitness function. However, since this model relies on statistical averaging, it is incapable of capturing the detailed coherent degradation of scattering fields under deterministic loads [22].

In summary, the existing research still exhibits the following deficiencies: (1) Most studies consider only a single structural deformation mechanism and have not yet incorporated the spatial displacements induced by aerodynamic pressure, the pointing deflections caused by the intrinsic curvature of the carrier, and the material efficiency degradation due to temperature rise into a unified theoretical framework. (2) For the superimposed effects of inherent geometric distortion of conformal carriers and service thermal loads, there is a lack of in-depth explanation of the physical mechanisms underlying the degradation of the scattering field phase distribution, and particularly, a quantitative analysis of the coupled effects of structural deformation and thermal effects on the scattering field remains insufficient. (3) Current scattering models rely on full-wave numerical simulations as an intermediate step, and an analytical and rapid mapping pathway from flight condition parameters to RCS responses has not yet been established.

To this end, this paper constructs a comprehensive electromechanical–thermal coupled scattering field model for conformal load-bearing antennas. By incorporating element-level geometric corrections, material corrections, and phase corrections in a stepwise superposition manner, the model establishes a complete solution from environmental loads to the scattering field RCS. The main contributions of this paper are as follows: (1) a complete electromechanical–thermal coupled mapping model from flight Mach number to array RCS is established, with a calculation error of less than 1 dB within the ±30° range; (2) decoupling analysis reveals that structural deformation is the dominant factor in RCS degradation, with the trailing-edge array experiencing an RCS peak increment of up to 7 dB at 0.65 Ma; (3) a BP neural network surrogate model is constructed, which significantly reduces the computational time for multi-physics field analysis, with a prediction relative error of less than 5%. However, the proposed model is based on the independent scattering approximation, which neglects mutual coupling among elements and edge effects. This approximation maintains good engineering accuracy when the element spacing is no less than half the wavelength and the array scale is relatively large. When the scan angle exceeds ±60°, the error increases significantly; therefore, the effective angular range of the model is ±60°.

## 2. Construction of the Electromechanical-Thermal Coupled Scattering Field Model for Conformal Load-Bearing Antennas

**Figure 1:**
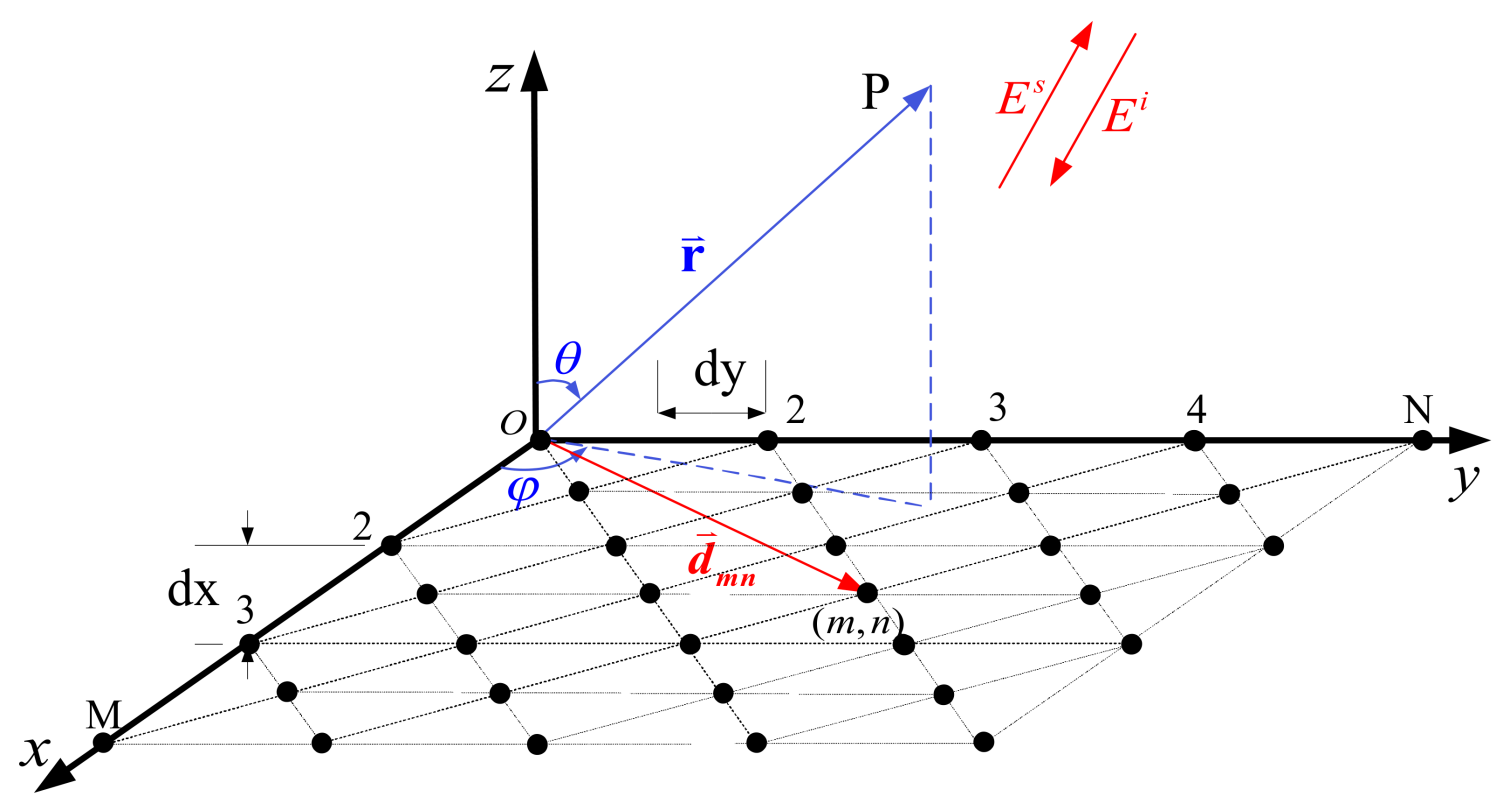
Schematic diagram of scattering from an array antenna.

The scattering characteristics of an array antenna are governed by both the scattering response of each individual element and the spatial distribution of the elements over the aperture plane, together with the associated interference effects. Under far-field plane-wave illumination, the total scattering field of the array can be expressed as the coherent superposition of the scattering fields of all elements, as illustrated in Figure 1.

For an aperture plane consisting of M×N elements located in the xoy plane, with its geometric center coinciding with the coordinate origin and the array normal oriented along the positive z-axis, let the incident wave direction be denoted by $\left(\theta_{i},\phi_{i}\right)$ and the scattered wave direction by $\left(\theta_{s},\phi_{s}\right)$. Then, the spatial phase difference in the (m,n)-th element with respect to the reference point is given by

(1) $$\Phi_{mn}^{\mathrm{rad}}=k\left(x_{mn}\sin\theta_{s}\cos\phi_{s}+y_{mn}\sin\theta_{s}\sin\phi_{s}\right)$$

For the scattering field, the incident and scattered waves experience two propagation paths; therefore, the spatial phase difference is twice that of the radiation case:

(2) $$\Phi_{mn}^{\mathrm{sca}}=k\left[x_{mn}(\sin\theta_{s}\cos\phi_{s}-\sin\theta_{i}\cos\phi_{i})+y_{mn}(\sin\theta_{s}\sin\phi_{s}-\sin\theta_{i}\sin\phi_{i})\right]$$

Under the assumption that mutual coupling between elements and edge effects of the array are neglected, the structural-mode scattering field of each array element is identical, denoted as ${\vec{E}}_{\mathrm{SM},0}$. Similarly, the antenna-mode scattering field of each element is identical, denoted as ${\vec{E}}_{\mathrm{AM},0}$. The total scattering field of the array can then be expressed as

(3) $${\vec{E}}_{\mathrm{array}}={\vec{E}}_{\mathrm{SM},0}\sum_{m=1}^{M}\sum_{n=1}^{N}e^{j\Phi_{mn}^{\mathrm{sca}}}+{\vec{E}}_{\mathrm{AM},0}\sum_{m=1}^{M}\sum_{n=1}^{N}e^{j\Phi_{mn}^{\mathrm{sca}}}$$

Equation (3) can be written in a compact form as

(4) $${\vec{E}}_{\mathrm{array}}={\vec{E}}_{\mathrm{element}}\cdot {\mathrm{AF}}_{\mathrm{sca}}$$

where ${\mathrm{AF}}_{\mathrm{sca}}$ is the scattering-field array factor, which is given by

(5) $${\mathrm{AF}}_{\mathrm{sca}}(\theta_{s},\phi_{s};\theta_{i},\phi_{i})=\sum_{m=1}^{M}\sum_{n=1}^{N}e^{jk(x_{mn}\Delta u+y_{mn}\Delta v)}$$

where $\Delta u=\sin\theta_{s}\cos\phi_{s}-\sin\theta_{i}\cos\phi_{i}$, $\Delta v=\sin\theta_{s}\sin\phi_{s}-\sin\theta_{i}\sin\phi_{i}$.

Accordingly, the total RCS of the array is given by

(6) $$\sigma_{\mathrm{array}}=\sigma_{\mathrm{element}}\cdot {\left|{\mathrm{AF}}_{\mathrm{sca}}\right|}^{2}$$

### 2.1. Geometric Distortion Induced by Conformal Mapping

The primary design characteristic of a conformal load-bearing antenna lies in its geometric configuration, which must conform to the aerodynamic contour of the wing skin. In this process, the originally regularly arranged ideal planar array undergoes a geometric transformation to be mapped onto a three-dimensional curved surface. This mapping procedure primarily introduces two types of geometric distortion: pointing deflection and bending deformation.

**Figure 2:**
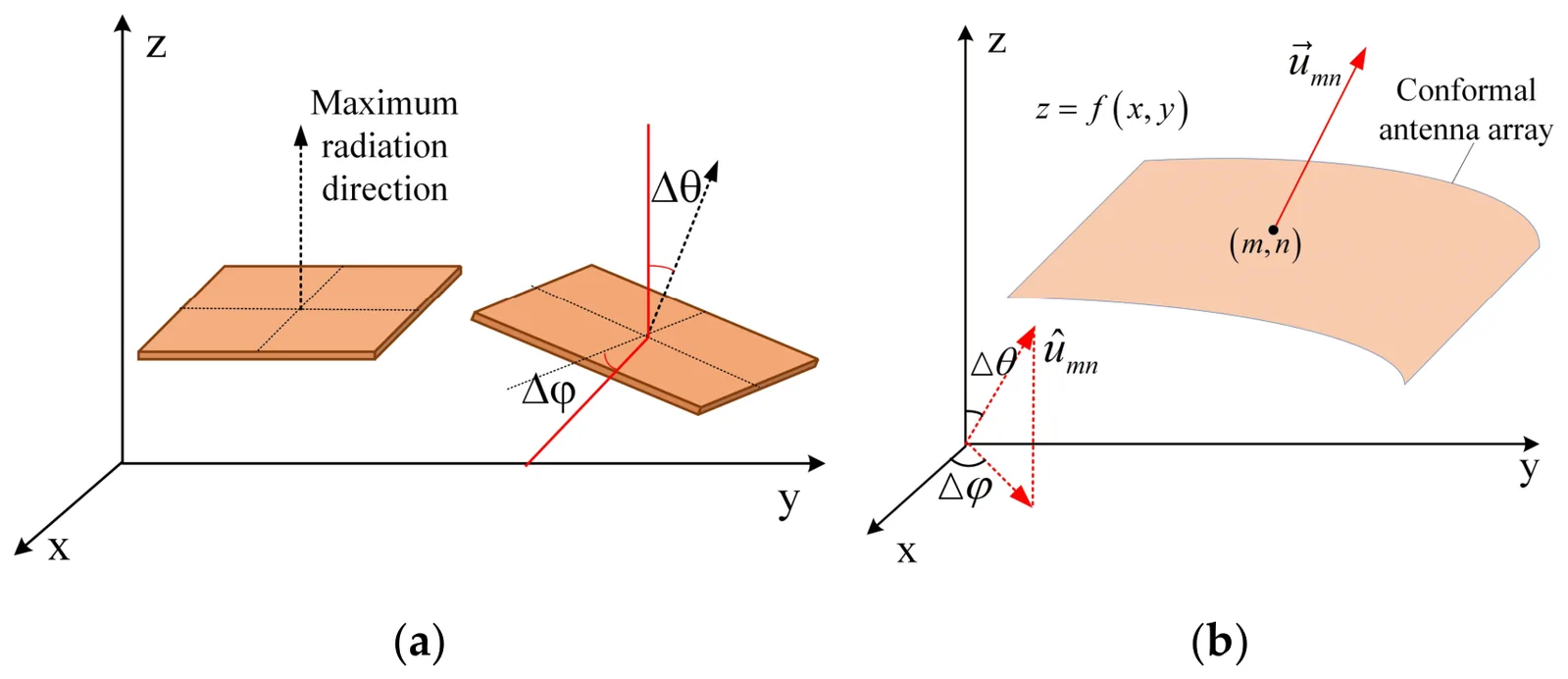
Pointing deflection of array elements and its determination. (**a**) Element pointing deflection. (**b**) Calculation of pointing deflection amount.

Pointing deflection refers to the change in the spatial orientation of individual elements when the planar array undergoes conformal mapping onto the curved surface of the carrier, as shown in Figure 2.

Let the normal vector of the surface at the i-th element be denoted as ${\vec{n}}_{i}=(n_{ix},n_{iy},n_{iz})$. Then, the deflection angle of this element relative to an ideal planar element (whose normal is along the z-axis) can be obtained from the angle between the normal vectors. The pitch deflection angle $\Delta \theta_{i}$ and the azimuth deflection angle $\Delta \phi_{i}$ of the element are respectively expressed as

(7) $$\Delta \theta_{i}=\arccos\left(\frac{n_{iz}}{\sqrt{n_{ix}^{2}+n_{iy}^{2}+n_{iz}^{2}}}\right)$$

(8) $$\Delta \phi_{i}=\arctan\left(\frac{n_{iy}}{n_{ix}}\right)$$

The corrected equivalent incident angle is given by

(9) $$\theta_{i}^{\prime }=\theta_{i}+\Delta \theta_{i}$$

(10) $$\phi_{i}^{\prime }=\phi_{i}+\Delta \phi_{i}$$

Substituting the corrected equivalent angles into Equation (2), the structural-mode RCS of the array element affected by pointing deflection is obtained as

(11) $$\sigma_{\mathrm{SM},i}=\frac{4\pi A^{2}}{\lambda^{2}}\cos^{2}\theta_{i}^{\prime }\cdot {\sin c}^{2}(kL\sin\theta_{i}^{\prime }\cos\phi_{i}^{\prime })\cdot {\sin c}^{2}(kW\sin\theta_{i}^{\prime }\sin\phi_{i}^{\prime })$$

Bending deformation denotes the modification of the boundary conditions of the electromagnetic field distribution inside the resonant cavity, which arises from the bending of the element substrate as the microstrip array conforms to a curved carrier. Although the physical arc lengths L and W of the microstrip patch remain constant, the effective electromagnetic radiation aperture in space is reduced due to bending. To address this, this paper adopts the equivalent structural dimension method, in which the projected chord length of the curved arc after bending is used to equivalently replace the physical structural dimensions of the element.

**Figure 3:**
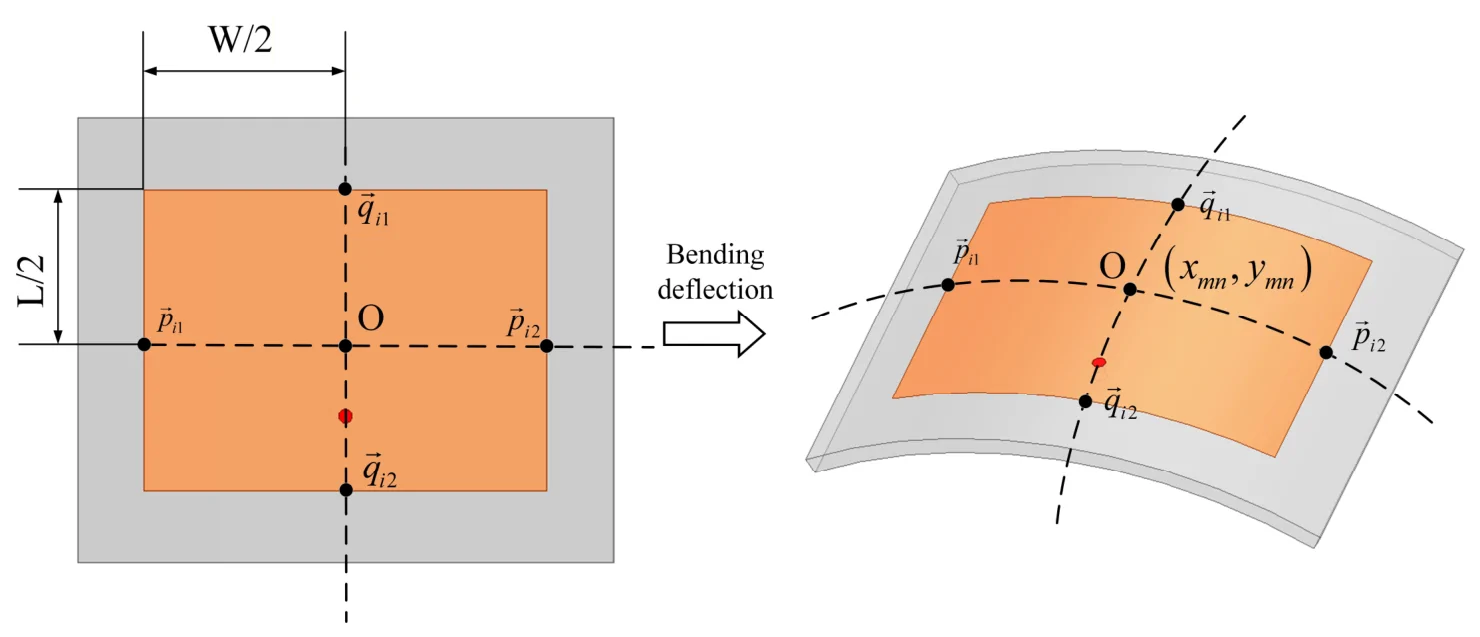
Schematic diagram of bending deformation of a rectangular microstrip patch antenna.

As shown in Figure 3, let the coordinates of the edge nodes of the i-th element along the length direction be ${\vec{p}}_{i1}$ and ${\vec{p}}_{i2}$, and those along the width direction be ${\vec{q}}_{i1}$ and ${\vec{q}}_{i2}$, respectively. Then the equivalent length and equivalent width are respectively given by

(12) $$L_{i}^{\prime }=\left\|{\vec{p}}_{i1}-{\vec{p}}_{i2}\right\|$$

(13) $$W_{i}^{\prime }=\left\|{\vec{q}}_{i1}-{\vec{q}}_{i2}\right\|$$

Accordingly, the structural dimension reduction ratio due to bending deformation is given by

(14) $$\delta L_{i}=\frac{L-L_{i}^{\prime }}{L}$$

(15) $$\delta W_{i}=\frac{W-W_{i}^{\prime }}{W}$$

Substituting the equivalent dimensions $L_{i}^{\prime }$ and $W_{i}^{\prime }$ into Equation (11), the structural-mode RCS of the array element, accounting for both pointing deflection and bending deformation, is obtained as

(16) $$\sigma_{\mathrm{SM},i}=\frac{4\pi {\left(L_{i}^{\prime }W_{i}^{\prime }\right)}^{2}}{\lambda^{2}}\cos^{2}\theta_{i}^{\prime }\cdot {\sin c}^{2}\left(kL_{i}^{\prime }\sin\theta_{i}^{\prime }\cos\phi_{i}^{\prime }\right)\cdot {\sin c}^{2}\left(kW_{i}^{\prime }\sin\theta_{i}^{\prime }\sin\phi_{i}^{\prime }\right)$$

### 2.2. Effects of Aerothermal Heating

In high-speed service conditions, aerodynamic heating is a critical physical-field factor that affects the performance of conformal antennas. The antenna efficiency η is defined as the ratio of the radiated power to the net input power, and can be expressed using the quality factor as

(17) $$\eta =\frac{1}{1+\frac{Q_{r}}{Q_{c}}+\frac{Q_{r}}{Q_{d}}+\frac{Q_{r}}{Q_{sw}}}$$

where $Q_{r}$, $Q_{c}$, $Q_{d}$ and $Q_{sw}$ denote the quality factors for radiation loss, conductor loss, dielectric loss, and surface-wave loss, respectively. For microstrip antennas, $Q_{sw}$ is usually much larger than $Q_{r}$; therefore, $\frac{Q_{r}}{Q_{sw}}$ is far smaller than the other terms and can be neglected.

The conductor loss quality factor $Q_{c}$ is proportional to the conductor conductivity $\sigma_{c}$. The conductivity of metals decreases with increasing temperature, and the approximate relationship is given by

(18) $$\sigma_{c}(T)=\frac{\sigma_{c0}}{1+\alpha \left(T-T_{0}\right)}$$

where $\sigma_{c0}$ denotes the conductivity at the reference temperature $T_{0}$, and α is the temperature coefficient of the metal. The temperature-modulated conductor loss quality factor is then given by

(19) $$Q_{c}(T)=Q_{c0}\cdot \frac{\sigma_{c}(T)}{\sigma_{c0}}=\frac{Q_{c0}}{1+\alpha \left(T-T_{0}\right)}$$

The dielectric loss quality factor $Q_{d}$ is inversely proportional to the dielectric loss tangent $\tan\delta_{d}$. The dielectric loss tangent of the dielectric material generally tends to increase with increasing temperature:

(20) $$\tan\delta_{d}(T)=\tan\delta_{d0}\cdot e^{\beta \left(T-T_{0}\right)}$$

where β denotes the temperature drift coefficient of the dielectric material. The dielectric loss quality factor is then given by

(21) $$Q_{d}(T)=Q_{d0}\cdot \frac{\tan\delta_{d0}}{\tan\delta_{d}(T)}=Q_{d0}\cdot e^{-\beta \left(T-T_{0}\right)}$$

Substituting Equations (19) and (21) into Equation (17), the temperature-modulated antenna efficiency is obtained as

(22) $$\eta (T)=\frac{1}{1+\frac{Q_{r}}{Q_{c0}}(1+\alpha (T-T_{0}))+\frac{Q_{r}}{Q_{d0}}e^{\beta (T-T_{0})}}$$

Substituting Equation (22) into the antenna-mode RCS expression (3), the AM-SF accounting for the temperature effect is obtained as

(23) $$\sigma_{\mathrm{AM},i}(T_{i})=\frac{\lambda^{2}}{4\pi }G_{i}^{2}(\theta_{i}^{\prime },\phi_{i}^{\prime })\cdot {\left|\Gamma_{i}\right|}^{2}\cdot \eta (T_{i})$$

where $T_{i}$ denotes the local equivalent temperature of the i-th element.

Within the temperature range of concern in this study, the variation in substrate permittivity $\varepsilon_{r}$ is generally less than 2%. The effects of resonant frequency shift and thermal-expansion-induced dimensional changes on antenna efficiency are far less significant than the temperature sensitivity of conductor and dielectric losses. Therefore, this paper primarily considers the temperature dependence of conductor conductivity and dielectric loss tangent, while neglecting the drift of permittivity and resonant frequency. The material parameters adopted are as follows: copper conductivity $\sigma_{c0}=5.8\times 10^{7}\;S/m$, with a temperature coefficient $\alpha =0.0039\;K^{-1}$; the substrate is Rogers 5880, with permittivity $\varepsilon_{r}=2.20\pm 0.02$, loss tangent $\tan\delta_{d}=0.0009$, and a permittivity temperature coefficient of approximately −125 ppm/°C. The radiation quality factor $Q_{r}$, conductor quality factor $Q_{c}$, and dielectric quality factor $Q_{d}$ are calculated according to classical microstrip antenna theory, and at the operating frequency of this study, they are taken as $Q_{r}=150$, $Q_{c}=500$ and $Q_{d}=300$, respectively.

### 2.3. Phase Errors Induced by Aerodynamic Loads

**Figure 4:**
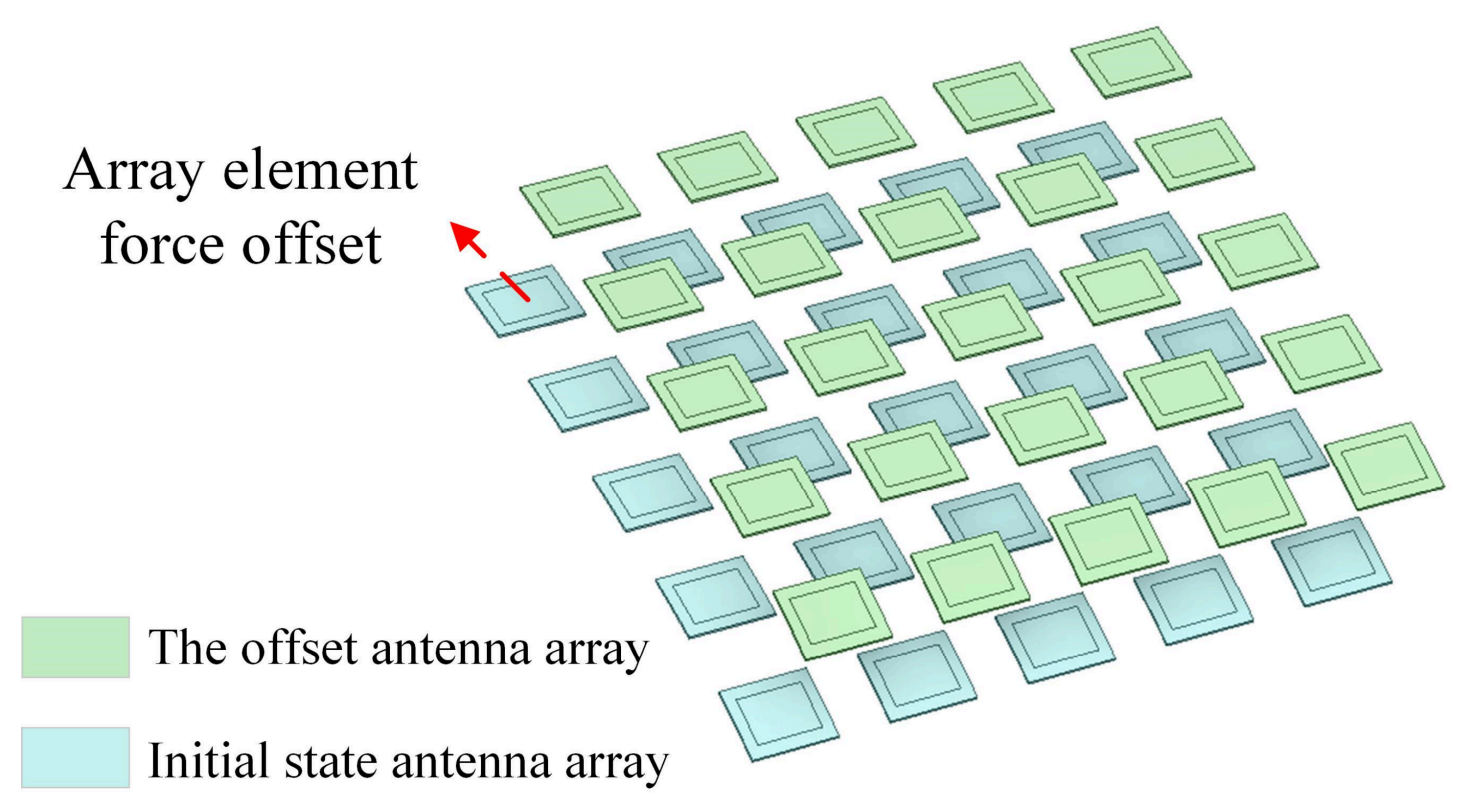
Schematic diagram of element position displacement under aerodynamic loads.

Under aerodynamic pressure, the wing skin undergoes significant macroscopic bending and torsional deflections, causing each microstrip element in the conformal array to deviate from its initial design position and resulting in non-uniform three-dimensional spatial translations, as shown in Figure 4.

Let the initial design position of the i-th element be denoted as ${\vec{r}}_{i}^{0}$,and its deformed position as ${\vec{r}}_{i}$. The position displacement vector is then given by

(24) $$\Delta {\vec{r}}_{i}={\vec{r}}_{i}-{\vec{r}}_{i}^{0}$$

For bistatic scattering, the additional phase difference introduced by the position displacement is given by

(25) $$\Delta \psi_{i}=k\left({\hat{r}}_{s}-{\hat{r}}_{i}\right)\cdot \Delta {\vec{r}}_{i}$$

where ${\hat{r}}_{s}$ is the unit vector in the scattering direction, and ${\hat{r}}_{i}$ is the unit vector in the incident direction. For monostatic scattering (${\hat{r}}_{s}=-{\hat{r}}_{i}$), Equation (25) simplifies to

(26) $$\Delta \psi_{i}=2k{\hat{r}}_{i}\cdot \Delta {\vec{r}}_{i}$$

### 2.4. Electromechanical–Thermal Coupled Scattering Field Model for Conformal Load-Bearing Antennas

For conformal load-bearing antennas, the pointing deflection, bending deformation, and local temperature vary from element to element, resulting in unit-dependent scattering fields $\sigma_{\mathrm{SM},i}$ and $\sigma_{\mathrm{AM},i}$ as well as their intrinsic phases $\psi_{\mathrm{SM},i}$ and $\psi_{\mathrm{AM},i}$. Consequently, a common factor cannot be extracted from the summation and the conventional array factor is no longer valid. Therefore, this paper directly constructs the vector superposition of scattering fields at the element level. By incorporating the correction factors derived in Section 2.1, Section 2.2 and Section 2.3, the total scattering field of the conformal load-bearing antenna can be expressed as

(27) $${\vec{E}}_{\mathrm{total}}(\theta ,\phi )=\sum_{i=1}^{N}\left[\sqrt{\sigma_{\mathrm{SM},i}}e^{j\psi_{\mathrm{SM},i}}+\sqrt{\sigma_{\mathrm{AM},i}}e^{j\psi_{\mathrm{AM},i}}\right]e^{j\Phi_{i}}e^{j\Delta \psi_{i}}$$

where $\Phi_{i}$ denotes the spatial phase difference in the i-th element in the ideal conformal state, $\Delta \psi_{i}$ is the additional phase induced by aerodynamic displacement, and $\psi_{\mathrm{SM},i}$ and $\psi_{\mathrm{AM},i}$ are the intrinsic phases of the SM-SF and AM-SF, respectively. Correspondingly, in the power domain, the total RCS of the array is given by

(28) $$\sigma_{\mathrm{total}}(\theta ,\phi )=\frac{4\pi }{\lambda^{2}}{\left\|{\vec{E}}_{\mathrm{total}}\right\|}^{2}$$

Equation (28) constitutes the electromechanical–thermal coupled scattering field model for conformal load-bearing antennas established in this paper. This model does not rely on a common array factor; the coordinate, pointing, temperature, and deformation effects of each element are independently incorporated into their respective phases and amplitudes.

## 3. Simulation and Validation

In this section, a cylindrical conformal array is first employed to validate the prediction accuracy of the proposed electromechanical–thermal coupled model. A BP neural network surrogate model is also constructed to enable rapid solution of the array physical fields. On this basis, the RCS evolution characteristics of the leading-edge, upper-surface, and trailing-edge arrays are analyzed over the Mach regime of 0.20–0.65 Ma. Furthermore, the respective contributions of structural deformation and temperature effects are decoupled to reveal the dominant mechanism by which aerodynamic loads affect the scattering performance.

### 3.1. Validation of the Electromechanical–Thermal Coupled Scattering Field Model

**Figure 5:**
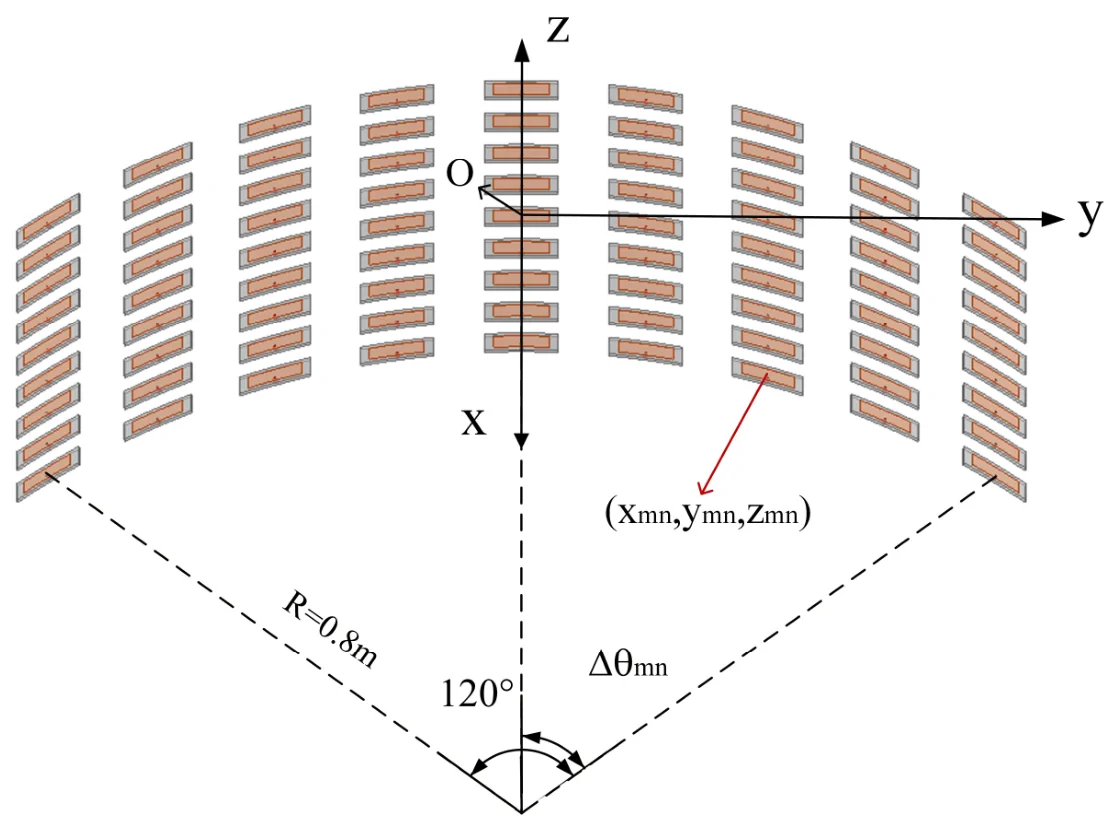
Structure of the cylindrical conformal load-bearing antenna.

To validate the effectiveness and accuracy of the proposed model, a cylindrical conformal array, as shown in Figure 5, is taken as an example for simulation. The array consists of 9 × 9 rectangular microstrip antenna elements arranged in a rectangular grid with both row and column spacings equal to 0.7λ. The antenna elements adopt the rectangular microstrip patch structure designed in Section 2. Owing to the attachment to a cylindrical carrier, the array surface exhibits a curved profile with a centrally raised convexity.

To validate the model, an ideal planar array is assumed to operate at a constant temperature of 25 °C. For the cylindrical conformal array, considering its thermal load characteristics, the temperature distribution over the array surface is assumed to remain constant along the x-axis direction, while exhibiting a centrally symmetric distribution along the y-axis direction. Specifically, the temperature reaches its maximum in the central region and gradually decreases toward both edges. The specific temperature distribution of each element is listed in Table 1.

**Figure 6:**
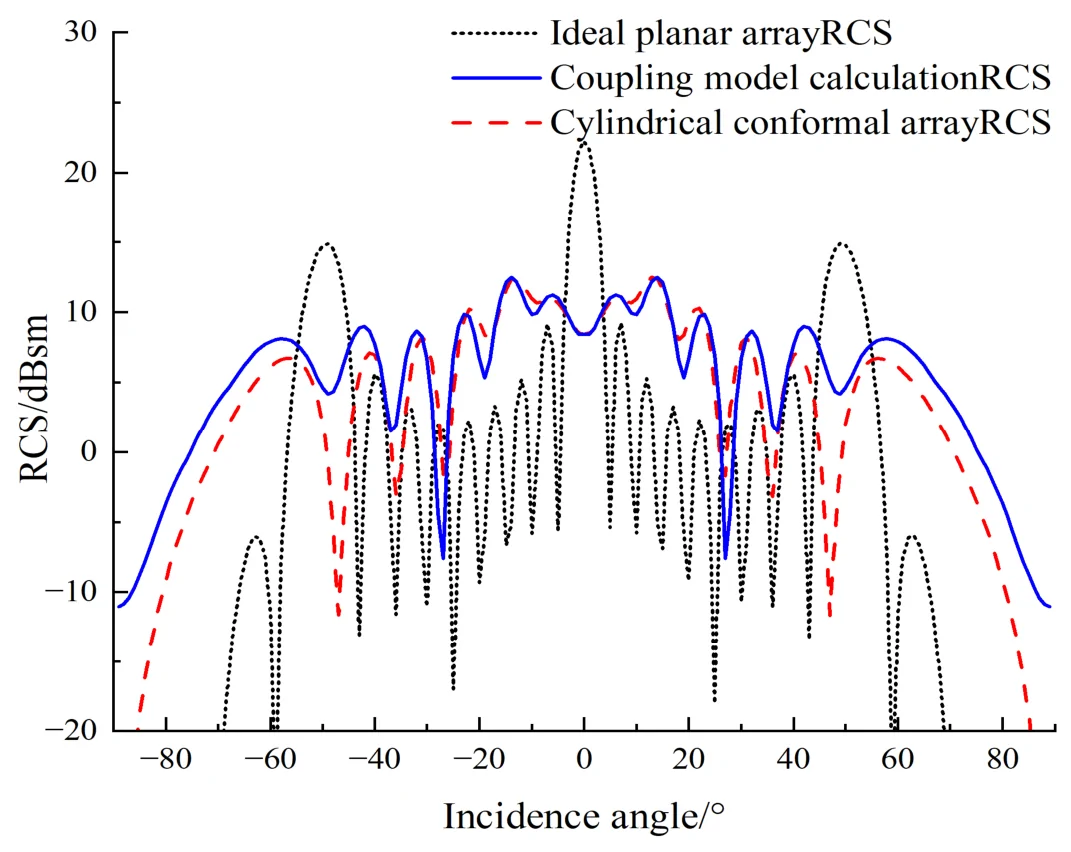
Scattering pattern of the cylindrical conformal array antenna.

The cylindrical radius is 0.8 m, and the center point of the element at the (5,5) position (the geometric center of the array) is selected as the origin of the global coordinate system. Under this coordinate definition, the pointing deflection of the elements in the central column is identically zero, while the pointing deflection of the entire array exhibits a centrosymmetric distribution with respect to the curvature. The position coordinates, bending deformation, pointing deflection, and temperature-induced efficiency variation of each element are computed and then substituted into the RCS coupled model for the conformal array antenna. The resulting scattering pattern is obtained and compared with the simulated RCS curves, as shown in Figure 6.

By comparing the HFSS-simulated characteristics of the ideal planar array and the conformal array, it is evident that the conformal structure significantly alters the energy distribution of the scattering field. The carrier curvature disrupts the phase planarity of the array scattering aperture, resulting in broadening of and notable reduction in the RCS peaks of both the main lobe and grating lobes. Specifically, the broadside RCS of the conformal array is 7.31 dBsm, which is reduced by 14.97 dB compared with that of the ideal planar array (22.28 dBsm). This indicates that the conformal structure can effectively suppress specular reflection in the broadside direction and offers a significant advantage in enhancing the stealth performance of the antenna.

Furthermore, the RCS curves computed by the coupled model are compared with the HFSS simulation results of the cylindrical conformal array. The results demonstrate that, within the range of approximately ±30°, the two are in excellent agreement, with the peak and null positions essentially coinciding and the absolute error maintained within 1 dB. When the scan angle exceeds the effective range of the model ($\left|\theta \right|>60^{\circ }$), the positions and amplitudes of the extreme points deviate due to the reduced accuracy of the independent scattering approximation. Nevertheless, the overall envelope trend of the scattering field across the full angular domain is still captured, thereby validating the effectiveness of the proposed electromechanical–thermal coupled model in predicting the scattering characteristics of conformal arrays under complex service environments.

### 3.2. Construction of Surrogate Model for Service Environment Prediction of Conformal Load-Bearing Antennas

**Figure 7:**
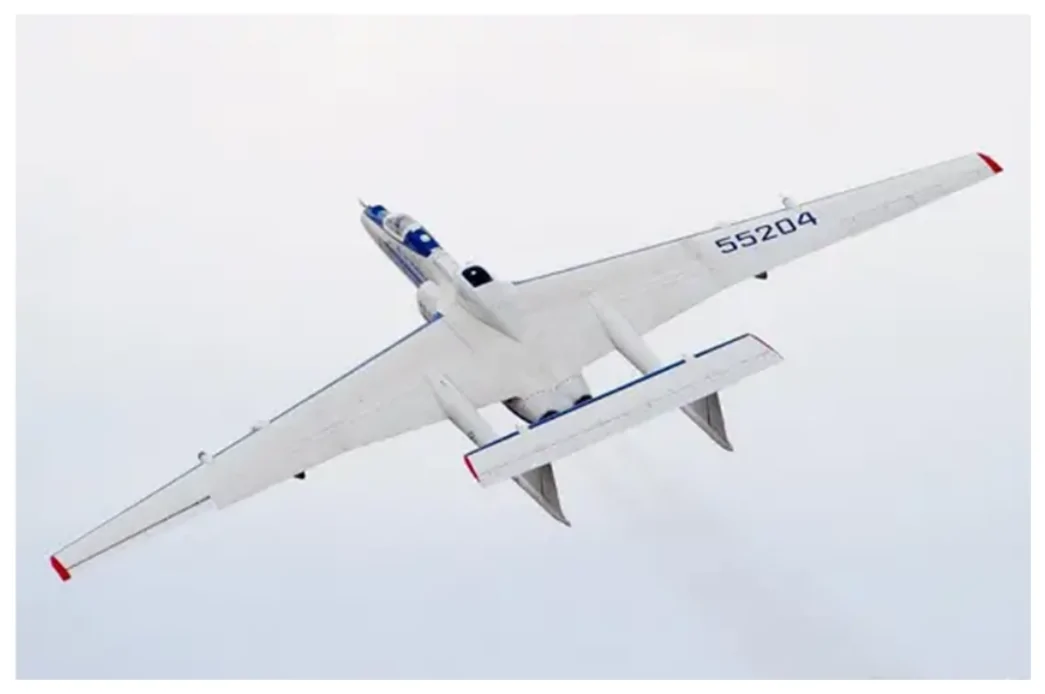
M-55 high-altitude reconnaissance aircraft.

**Figure 8:**
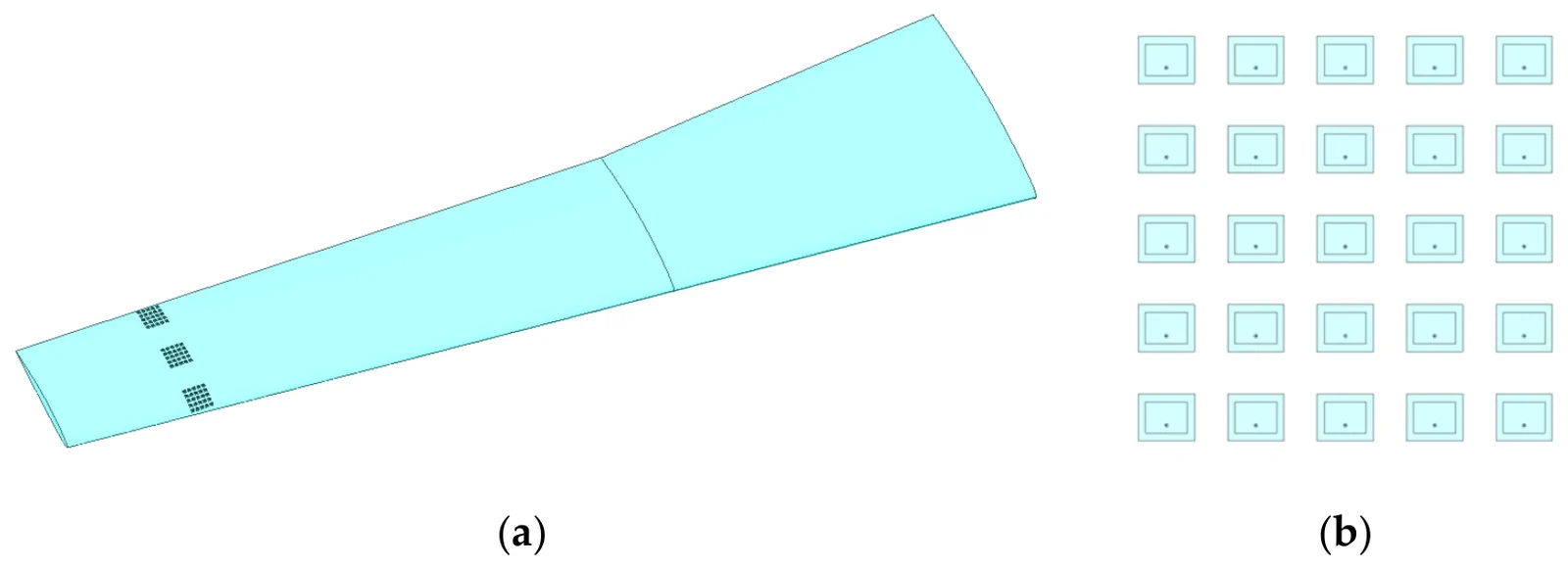
Schematic diagram of the wing-integrated conformal array antenna layout. (**a**) Overall layout of the wing-integrated conformal array. (**b**) Local details of the antenna array.

Although finite element numerical simulation can compute the structural response of the wing and antenna array with high accuracy, a single computation involves multiple sequential steps, including flow-field iteration, data mapping, and structural solving, resulting in considerable computational time. To enable rapid parametric evaluation from loading conditions to the corresponding electrical performance response, a surrogate model for predicting the service environment of conformal load-bearing antennas is constructed in this paper. The wing of the M-55 high-altitude reconnaissance aircraft, as shown in Figure 7, is adopted as the research object. This aircraft features a high cruise altitude, long endurance, and an aerodynamic configuration characterized by a high aspect ratio. Its slender wing structure is highly sensitive to aerodynamic and thermal loads, making it an ideal platform for validating the performance variations of CLBAs under complex coupled mechanical–thermal environments. For the wing-integrated conformal array antenna shown in Figure 8, ten representative operating conditions are selected for simulation, with the specific configurations detailed in Table 2.

The flow field is computed using ANSYS 2024R1 Fluent by solving the steady compressible Reynolds-averaged Navier–Stokes equations with the SST k-ω turbulence model. The inlet is set as a velocity inlet, the outlet as a pressure outlet, and the wall is modeled as a no-slip adiabatic wall. Structural analysis is performed using ANSYS Mechanical, with the wing material specified as aluminum alloy. The fluid–structure coupling is achieved through a one-way coupling scheme in Workbench, where the surface pressure field and heat flux are mapped onto the structural mesh. The mesh consists of approximately 1.2 million elements, and all grids satisfy the boundary layer requirements. The temperature field is solved using a sequential coupling approach, in which the flow temperature is first computed and then applied as a thermal load to the structure. The computations are carried out on a 12th Gen Intel(R) Core(TM) i5-12400 computer, with each operating condition requiring approximately 2 h of computation time. Under the above-mentioned settings, finite element simulations are performed for ten typical operating conditions within the Mach range of 0.20 to 0.65 Ma. From the leading-edge antenna array, 1000 representative grid nodes are selected, yielding 10,000 sets of sample data comprising “mechanical–thermal loads and spatial positions.” A BP neural network with four hidden layers is constructed [23,24,25]. The input layer takes the Mach number and spatial coordinates (x, y, z) as inputs, while the output layer provides the node displacement and temperature. The numbers of neurons in the hidden layers are set to 10, 20, 20, and 10, respectively, with the Sigmoid activation function employed in the hidden layers and a Linear function used in the output layer. Weight updates are performed using the scaled conjugate gradient algorithm with a learning rate of 0.1. The samples are randomly divided into training, validation, and test sets in a ratio of 7:2:1, with the nodes corresponding to each operating condition number kept as an integral group during the partitioning process, and the model performance is evaluated using the mean absolute error (MAE), root mean square error (RMSE), and coefficient of determination (R^2^).

(29) $$MAE=\frac{1}{n}\sum_{i=1}^{n}|y_{i}-{\hat{y}}_{i}|$$

(30) $$RMSE=\sqrt{\frac{\sum_{i=1}^{n}{\left(y_{i}-{\hat{y}}_{i}\right)}^{2}}{n}}$$

(31) $$R^{2}=1-\frac{\sum {\left(y_{i}-{\hat{y}}_{i}\right)}^{2}}{\sum {\left(y_{i}-\bar{y}\right)}^{2}}$$

**Figure 9:**
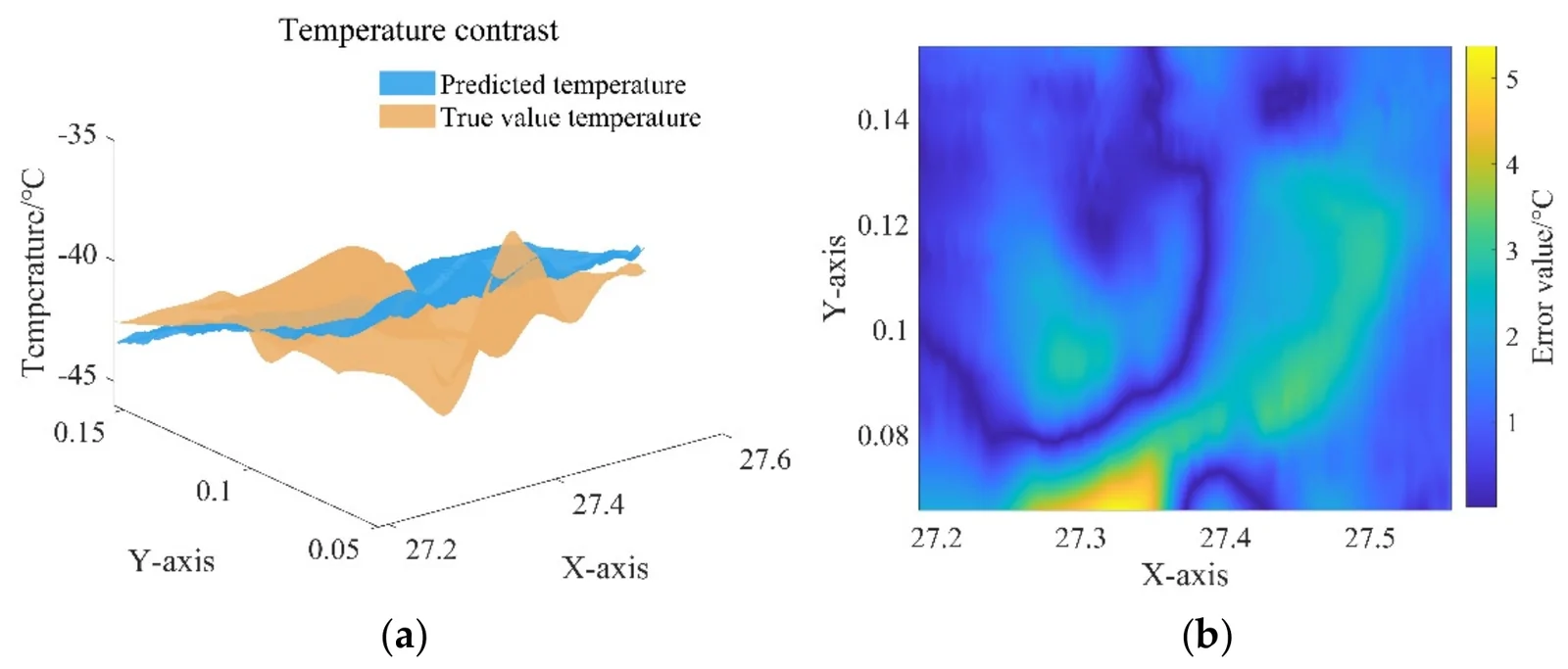

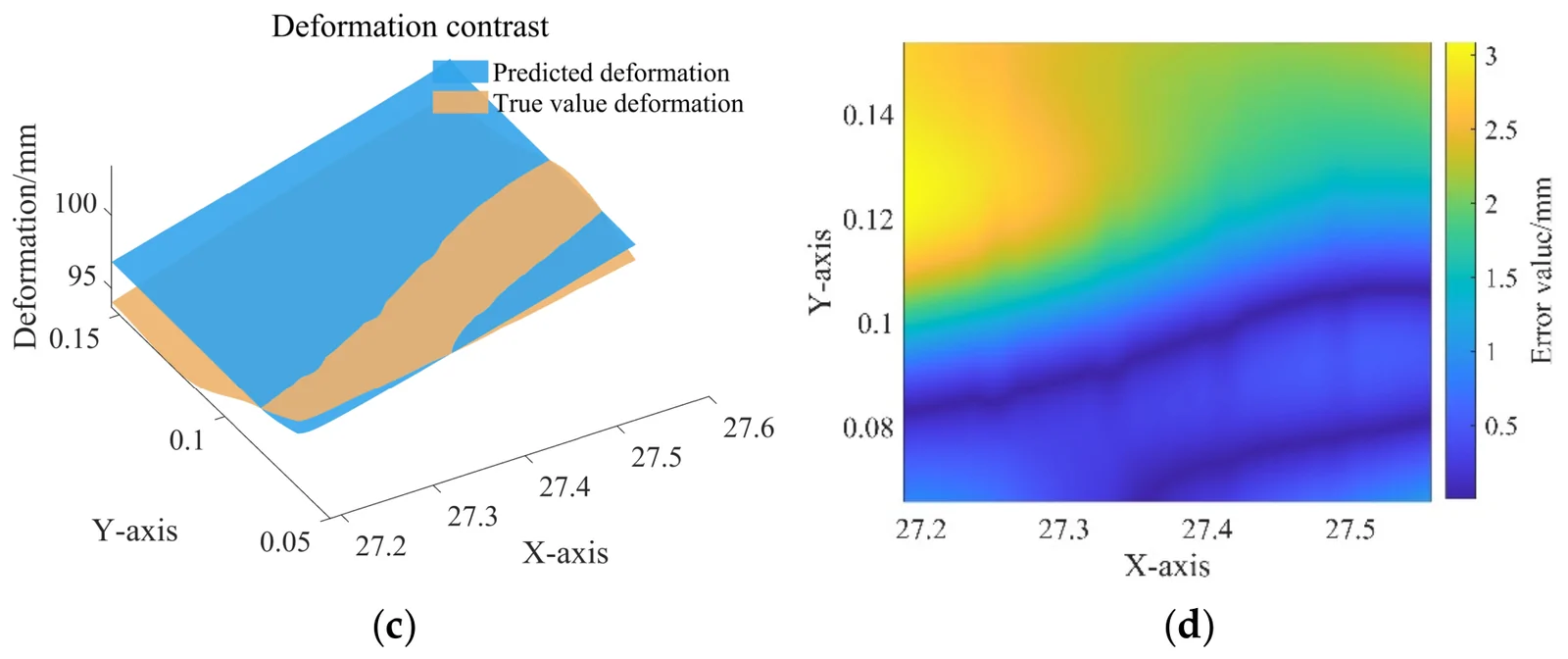
Validation of temperature and deformation predictions for the leading-edge antenna at 0.70 Ma. (**a**) Comparison of predicted temperature with true-value surface. (**b**) Contour map of temperature prediction error distribution. (**c**) Comparison of predicted deformation with true-value surface. (**d**) Contour map of deformation prediction error distribution.

Upon iterative training, the array deformation prediction model converges. The statistical results of model accuracy are presented in Table 3, indicating that the model achieves a high fitting accuracy. To further evaluate the generalization performance of the model across the continuous Mach regime, a validation case at 0.70 Ma (which lies outside the training range) is selected, and the predicted results are shown in Figure 9.

Under the operating condition of 0.70 Ma, the maximum prediction errors for temperature and deformation are approximately 6 °C and 3.5 mm, respectively, with the relative error controlled within 5%. The relative error is defined as

(32) $$RE=\frac{1}{n}\sum_{i=1}^{n}\frac{\left|y_{i}-{\hat{y}}_{i}\right|}{{\hat{y}}_{i}}$$

The predicted surfaces accurately reproduce the physical trend that the wingtip bending–torsion deformation increases significantly with increasing flow velocity. This demonstrates that the model is capable of providing physically plausible trend predictions even under conditions lacking prior information. Based on this surrogate model, rapid computation from loading conditions to deformation fields can be achieved, thereby enabling the scattering performance analysis of wing-integrated conformal load-bearing antennas under service environments.

### 3.3. Scattering Performance Analysis of Wing-Integrated Conformal Load-Bearing Antennas Under Service Environments

Under complex service environments, the scattering performance evaluation of wing-integrated conformal load-bearing antennas relies on the real-time and accurate awareness of the physical states of the array aperture. The electromechanical–thermal coupled scattering field model established in Section 2.4 requires the spatial position coordinates, pointing deflection angles, and real-time temperatures of each array element as input parameters. A 5 × 5 antenna array on the wing leading edge under the flight condition of 0.70 Ma is taken as an example for analysis, where the elements are arranged in a grid layout along the chordwise and spanwise directions: rows 1 to 5 represent the progression along the chordwise direction from the region away from the leading edge toward the stagnation point, while columns 1 to 5 are arranged along the spanwise direction from the distal wingtip toward the proximal wing root. The grid node corresponding to the (m,n) element is designated as element i. The data of the 5 × 5 leading-edge array nodes under the 0.70 Ma condition are shown in Table 4.

**Figure 10:**
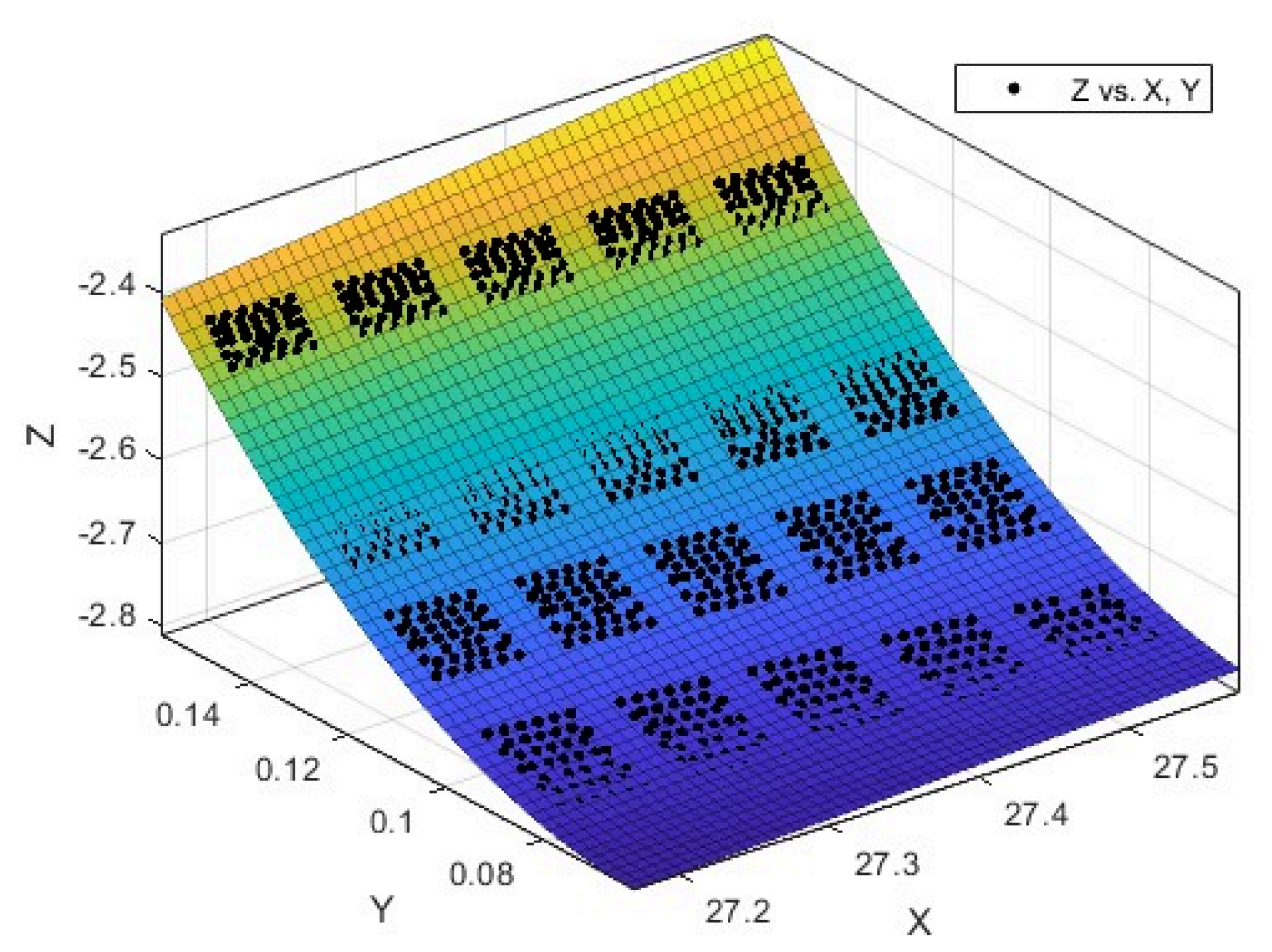
Surface fitting result of the leading-edge antenna array under the operating condition of 0.70 Ma.

Based on the spatial coordinates of each element grid node under service conditions, surface fitting is performed using the least-squares method to obtain the deformed array surface function. The fitting result is shown in Figure 10, and its expression is given as follows:

(33) $$\begin{array}{c} f(x,y)=4.3178-0.4982x-39.7133y\\ \;\;\;\;\;\;\;\;+0.0089x^{2}+1.2859xy+40.8027y^{2} \end{array}$$

Based on the fitted array surface, the nodal temperature information within the region of each element is further extracted. Owing to the complex flow-field distribution over the wing surface, the temperatures at different grid nodes within the same element are not uniform. To facilitate the subsequent scattering field calculation, the regional averaging method is adopted in this paper to determine the equivalent operating temperature of each element, as follows:

(34) $$T_{m,n}^{\prime }=\frac{1}{N}\sum_{j=1}^{N}T_{j}^{\prime }$$

where $T_{m,n}^{\prime }$ denotes the equivalent average temperature of the (m,n)-th element; N is the total number of grid nodes within the region covered by the (m,n)-th element; and $T_{j}^{\prime }$ is the predicted temperature at the corresponding node. Table 5 shows the equivalent average temperature distribution of the leading edge array elements under the 0.70 Ma condition.

**Figure 11:**
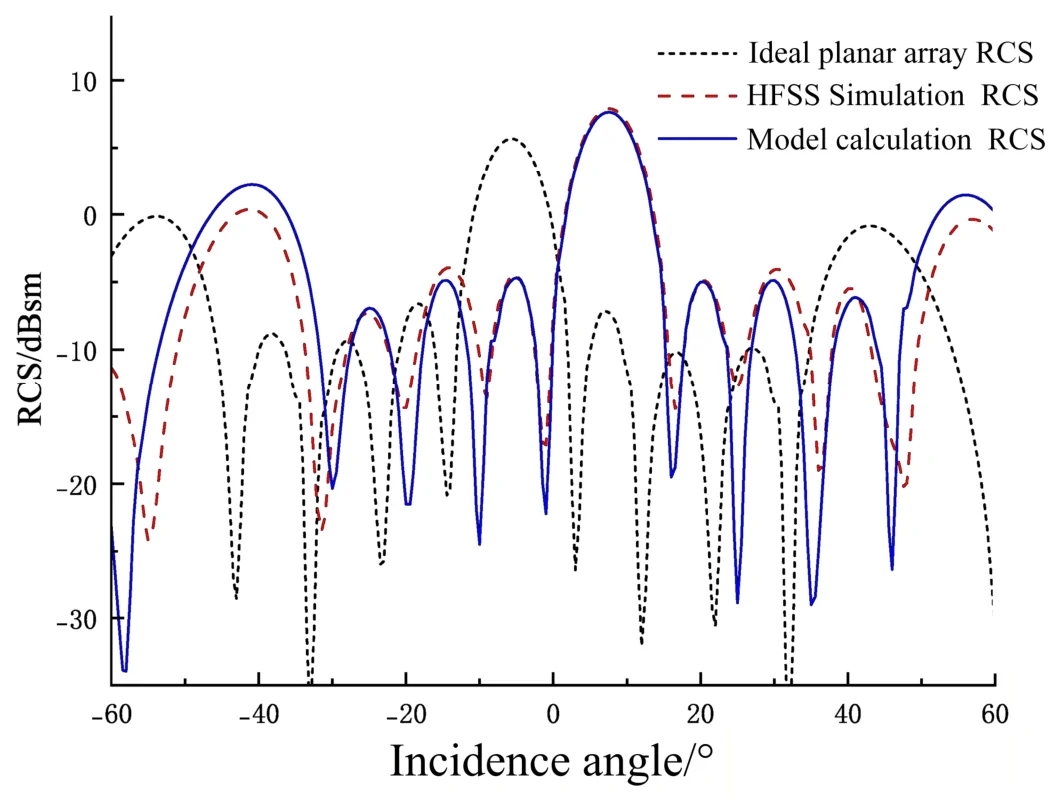
Numerical data of RCS scattering patterns under the operating condition of 0.70 Ma.

Based on the fitted array surface function and the nodal coordinates under service conditions, the pointing deflection angles and bending deformations of each element are accurately calculated. These structural parameters, together with the equivalent average temperatures, are then substituted into the electromechanical–thermal coupled scattering field model for conformal load-bearing antennas to obtain the RCS of the conformal array under the flight condition of 0.70 Ma. To evaluate the influence mechanism of environmental loads on the scattering performance of the wing-integrated conformal load-bearing antenna and to further verify the reliability of the model, the computed RCS is compared with the HFSS simulation results, with the RCS of the ideal planar array before deformation serving as the baseline reference. The comparison results are presented in Figure 11.

From the comparison results shown in Figure 11, it can be observed that the RCS curves computed by the proposed coupled model are in good agreement with the HFSS simulation results in terms of overall trend, which further validates the computational accuracy and reliability of the model at 0.70 Ma. By comparing the RCS of the planar array with that computed by the proposed model, it is evident that after the array evolves from the initial ideal planar state to the in-service conformal state, the leading-edge conformal antenna exhibits typical performance degradation characteristics, including increased mainlobe amplitude, beam-pointing deviation, and asymmetric sidelobe elevation, owing to the coupled effects of structural deformation and temperature variations. These results reveal the underlying mechanism by which aerodynamic loads affect the scattering performance of conformal load-bearing antennas.

### 3.4. Analysis of the Influence of Aerodynamic Loads on Scattering Performance

Section 3.3 has established and validated the scattering field calculation method based on the service environment, enabling the efficient coupled solution of the antenna array structural deformation, temperature distribution, and scattering field under arbitrary flight conditions. On this basis, the present section combines the physical-field data of ten typical cruise conditions (0.20–0.65 Ma) predicted by the surrogate model with the electromechanical–thermal coupled scattering field model. The influence of aerodynamic loads on the scattering performance of conformal load-bearing antennas is systematically revealed from three perspectives: the variation in flight Mach number, decoupling analysis of structural deformation and temperature effects, and array layouts at different wing positions.

**Figure 12:**
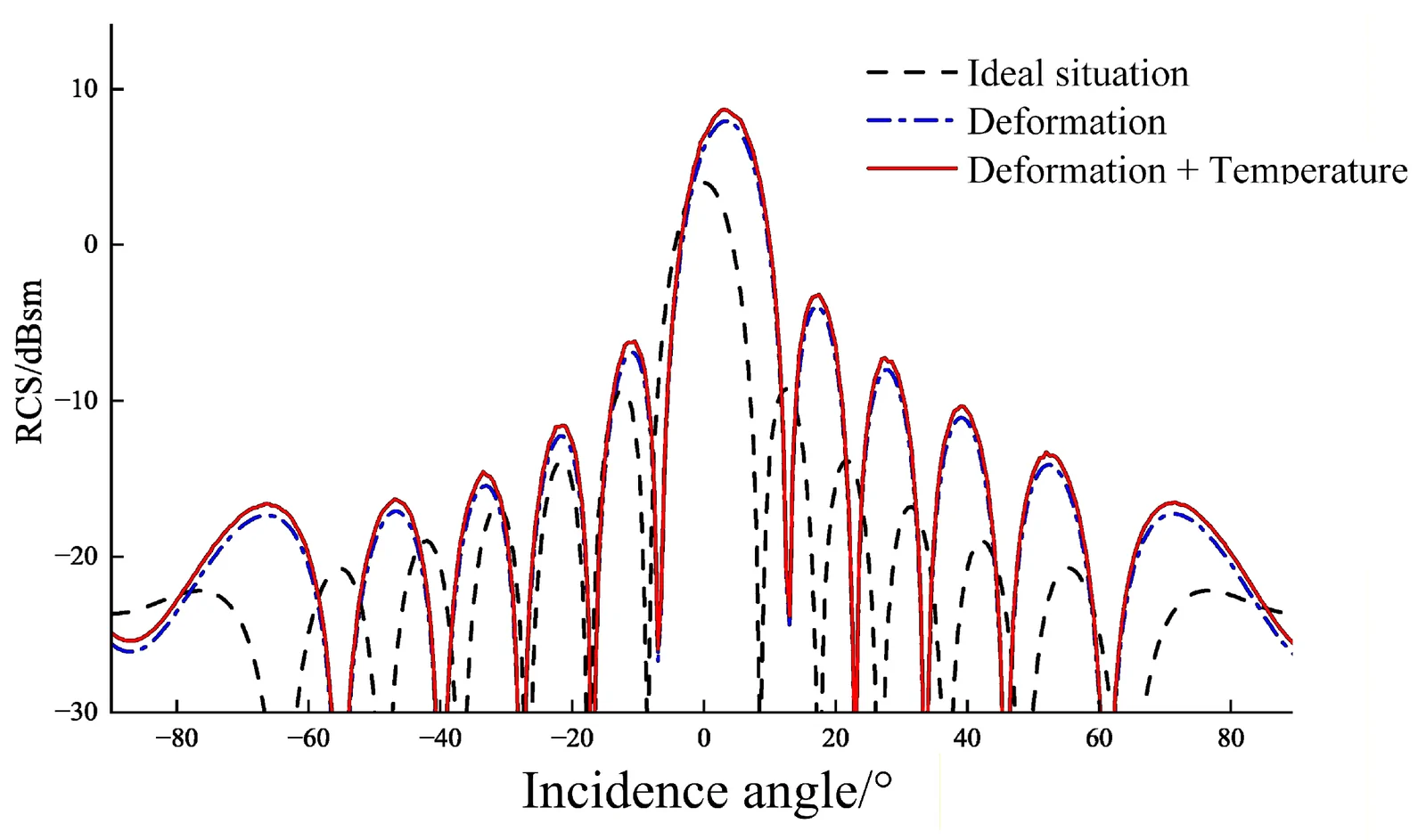
Decoupling analysis of structural deformation and thermal effects on the RCS of the leading-edge array under the operating condition of 0.65 Ma.

To distinguish the individual contributions and the coupled effects of structural deformation and temperature effects on scattering performance, a decoupling analysis is conducted using the wing leading-edge array under the condition of 0.65 Ma as the study object. The RCS characteristics are separately calculated for three states: considering structural deformation only, considering both structural deformation and temperature effects, and the ideal conformal antenna without deformation or temperature rise. The comparison results are presented in Figure 12.

From the three curves in Figure 12, it can be seen that at an incidence angle of 0°, the RCS peak in the ideal conformal state is approximately 5 dBsm, whereas it is approximately 9 dBsm when only structural deformation is considered, and approximately 9.5 dBsm when deformation and temperature effects are coupled. The additional contribution of the temperature effect is approximately 0.5 dB, which is much smaller than the contribution of structural deformation. Therefore, structural deformation is the dominant factor in RCS degradation, while the influence of temperature effects is relatively small but not negligible.

**Figure 13:**
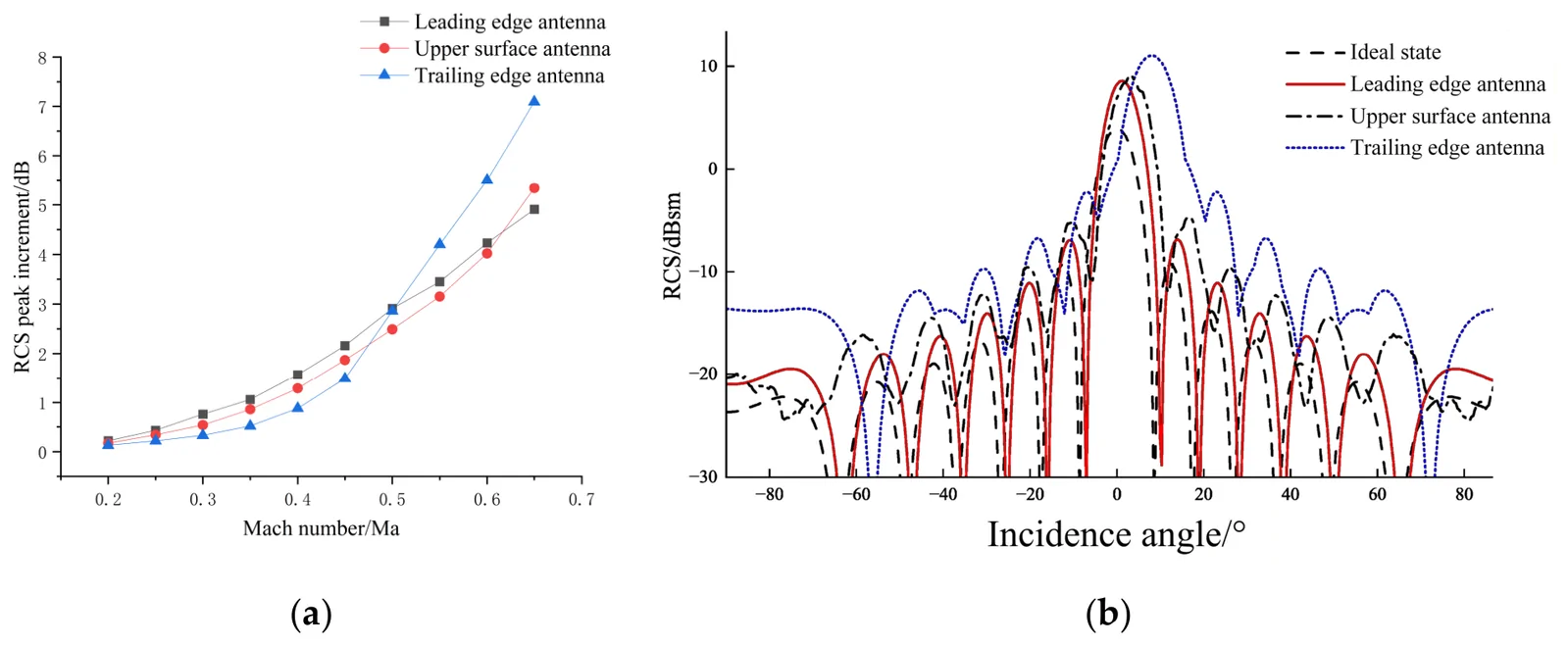
Evolution of scattering performance and waveform comparison for arrays at different layout positions. (**a**) RCS peak increment versus Mach number. (**b**) Comparison of RCS scattering patterns at 0.65 Ma.

Owing to the spatial variability in aerodynamic load distribution, structural stiffness, and temperature fields over the wing surface, the conformal arrays located at the leading edge, upper surface, and trailing edge experience distinct service environments and boundary conditions, resulting in significantly differentiated sensitivity of scattering performance to flight Mach number. To quantitatively characterize this evolution pattern, the increments in RCS peak values of the three array groups relative to the ideal conformal state across the full Mach regime from 0.20 Ma to 0.65 Ma, as well as the RCS scattering patterns of each array under the extreme condition of 0.65 Ma, are computed. The corresponding results are presented in Figure 13.

From Figure 13a, it can be observed that as the flight Mach number increases, the RCS peak increments of the conformal antenna arrays at the leading edge, upper surface, and trailing edge all exhibit a nonlinear upward trend. Among them, the leading-edge array experiences relatively small deformation, and its RCS increment shows an approximately linear and steady growth with increasing Mach number. The upper-surface array, located in the flow expansion region, exhibits RCS increment variations that consistently lie between those of the leading-edge and trailing-edge arrays. The trailing-edge array, being more significantly affected by wing bending–torsion deformation, undergoes larger relative deformation and exhibits pronounced non-uniformity in temperature distribution; its RCS increment increases rapidly when Ma > 0.50, ultimately reaching a peak increment of approximately 7 dB.

Combined with the scattering patterns at 0.65 Ma shown in Figure 13b, it is evident that the leading-edge array primarily exhibits an increase in the mainlobe peak value with a relatively small beam-pointing deviation of about 3°. The upper-surface array experiences a significant beam shift of approximately 8°, severely deviating from the nominal normal-direction design reference. The trailing-edge array, in contrast, is characterized by notable broadening of the scattering mainlobe and severe fluctuations in sidelobe levels. The distinct distortion features of the three array patterns further corroborate the differentiated evolution of scattering performance across different regions of the wing.

## 4. Conclusions

In this paper, to address the scattering performance evaluation of conformal load-bearing antennas under the coupled effects of aerodynamic loads and thermal effects, a comprehensive electromechanical–thermal coupled scattering field model is established, integrating geometric distortion corrections, temperature-dependent efficiency corrections, and spatial phase error corrections. The following main conclusions are drawn:

(1) An electromechanical–thermal coupled scattering field model for conformal load-bearing antennas is constructed, which reveals the modulation mechanism of conformal mapping on the structural-mode scattering field, the sensitivity relationship of temperature on antenna efficiency, and the degradation mechanism of array displacement on the scattering phase. A complete mapping chain from flight conditions to the array RCS is thereby established.
(2) Validation using a 9 × 9 cylindrical conformal array demonstrates that, within the scanning range of ±30°, the model calculations agree with HFSS simulations with an absolute error of less than 1 dB. Within the ±60° range, the model effectively captures the main envelope characteristics of the scattering pattern. Compared with the planar array, the broadside RCS of the conformal array is reduced by 14.97 dB, verifying the effectiveness of the proposed model.
(3) Analysis over the Mach regime of 0.20–0.65 Ma reveals that structural deformation is the dominant factor causing RCS distortion, followed by temperature effects, which are secondary but non-negligible. At 0.65 Ma, the RCS peak increment of the trailing-edge array reaches 7 dB, exhibiting a rapid nonlinear increase. The leading-edge array exhibits a mainlobe shift of approximately 3°, the upper-surface array shifts by about 8°, while the trailing-edge array is characterized by mainlobe broadening and severe sidelobe fluctuations.

In summary, the electromechanical–thermal coupled scattering field model developed in this paper enables the efficient prediction of multi-field coupled performance for conformal load-bearing antennas. The above work lays a theoretical and methodological foundation for the stealth performance evaluation of conformal antennas under complex service environments. Future work will consider the time-varying effects of mutual coupling among elements during the deformation process of large-curvature carriers, with the aim of further improving model accuracy under coupled conditions by introducing mutual coupling correction terms.

## Figures and Tables

**Table 1:** Temperature distribution of elements in the 9 × 9 cylindrical conformal array (unit: °C).

Row/ Column	1	2	3	4	5	6	7	8	9
1	35.2	38.5	41.2	45.5	48.0	45.5	41.2	38.5	35.2
2	35.2	38.5	41.2	45.5	48.0	45.5	41.2	38.5	35.2
3	35.2	38.5	41.2	45.5	48.0	45.5	41.2	38.5	35.2
4	35.2	38.5	41.2	45.5	48.0	45.5	41.2	38.5	35.2
5	35.2	38.5	41.2	45.5	48.0	45.5	41.2	38.5	35.2
6	35.2	38.5	41.2	45.5	48.0	45.5	41.2	38.5	35.2
7	35.2	38.5	41.2	45.5	48.0	45.5	41.2	38.5	35.2
8	35.2	38.5	41.2	45.5	48.0	45.5	41.2	38.5	35.2
9	35.2	38.5	41.2	45.5	48.0	45.5	41.2	38.5	35.2

**Table 2:** Ten representative operating conditions.

Condition No.	Mach Number (Ma)	Flow Velocity (m/s)	Condition No.	Mach Number (Ma)	Flow Velocity (m/s)
Case 1	0.20	59.0	Case 6	0.45	132.8
Case 2	0.25	73.8	Case 7	0.50	147.5
Case 3	0.30	88.5	Case 8	0.55	162.3
Case 4	0.35	103.3	Case 9	0.60	177.0
Case 5	0.40	118.0	Case 10	0.65	191.8

**Table 3:** Accuracy statistics of the surrogate model.

Predicted Physical Quantity	R^2^	RMSE	MAE
Structural deformation field	0.99998	0.0048	0.0039 mm
Temperature field	0.99993	0.0051	0.0042 °C

**Table 4:** Node data of the 5 × 5 leading-edge array under the operating condition of 0.70 Ma.

Element Grid Node (m,n,i)	Initial Coordinates (x,y,z)/m	Predicted Coordinates $\left(x^{\prime },y^{\prime },z^{\prime }\right)$/m	Initial Temperature T/°C	Predicted Temperature $T^{\prime }$/°C
(1, 1, 21)	(27.531, 0.076, −2.769)	(27.518, 0.079, −2.635)	−56.50	−43.85
(1, 5, 159)	(27.213, 0.076, −2.769)	(27.209, 0.077, −2.668)	−56.50	−44.13
(3, 3, 541)	(27.372, 0.124, −2.610)	(27.365, 0.128, −2.505)	−56.50	−41.35
(5, 1, 640)	(27.531, 0.151, −2.451)	(27.522, 0.156, −2.322)	−56.50	−38.47
(5, 5, 983)	(27.531, 0.151, −2.451)	(27.211, 0.152, −2.385)	−56.50	−38.62

**Table 5:** Equivalent average temperature distribution of the leading-edge array elements under the operating condition of 0.70 Ma.

Row/ Column	1	2	3	4	5
1	−43.85	−43.92	−44.05	−44.08	−44.13
2	−42.60	−42.75	−42.82	−42.85	−42.90
3	−41.15	−41.28	−41.35	−41.42	−41.50
4	−39.80	−39.95	−40.02	−40.15	−40.25
5	−38.47	−38.52	−38.58	−38.60	−38.62

## Data Availability

The original contributions presented in this study are included in the article. Further inquiries can be directed to the corresponding authors.

## References

[1] Li Z., Wang K., Lv Y., Qian S., Zhang X., Cui X. (2023). Wing conformal load-bearing endfire phased array antenna skin technology. IEEE Trans. Antennas Propag..

[2] Beziuk G., Krajewski A., Baum T.C., Nicholson K.J., Ghorbani K. (2024). Electromagnetic and electronic aerospace conformal load-bearing smart skins: A review. IEEE J. Microw..

[3] Fernandez M.G., Lopez Y.A., Arboleya A., González-Valdés B., Rodríguez-Vaqueiro Y., Gómez M.E.D.C., Andrés F.L.-H. (2017). Antenna diagnostics and characterization using unmanned aerial vehicles. IEEE Access.

[4] Hao S., Ma T., Wang Y., Li H., Zhao S., Tan P. (2023). Gust alleviation by active–passive combined control of the flight platform and antenna array for a flying wing SensorCraft. Aerospace.

[5] Lee C.Y., Kim J.H. (2021). Active flutter suppression of smart-skin antenna structures with piezoelectric sensors and actuators. Aerospace.

[6] Hao D., Liu J., He Y., Ding Z., Su J., Yang D. (2024). Optimization of MTI structure based on material characteristics under extreme aerodynamic heating environment. Therm. Sci. Eng. Prog..

[7] Feng S.Z., Sun Q.J., Xiao S., Han X., Li Y., Li Z. (2024). A novel multi-physics coupling model for the stochastic analysis of phased arrays considering material spatial uncertainty. Appl. Math. Model..

[8] Yang K., Zhao Z., Ouyang J., Nie Z., Liu Q. (2012). Optimisation method on conformal array element positions for low sidelobe pattern synthesis. IET Microw. Antennas Propag..

[9] Huang Z., Zhou J., Zhang H. (2015). Full polarimetric sum and difference patterns synthesis for conformal array. Electron. Lett..

[10] Yu T., Yin C., Liu H. (2015). Full wave analysis of the arbitrarily arranged spherical conformal microstrip antenna array. IET Microw. Antennas Propag..

[11] Green R. (1963). The General Theory of Antenna Scattering. Ph.D. Thesis.

[12] Munk B.A. (2003). Finite Antenna Arrays and FSS.

[13] Steyskal H. (2010). On the power absorbed and scattered by an antenna. IEEE Antennas Propag. Mag..

[14] Ossowska A., Kim J.H., Wiesbeck W. Influence of mechanical antenna distortions on the performance of the HRWS SAR system. Proceedings of the 2007 IEEE International Geoscience and Remote Sensing Symposium (IGARSS).

[15] Zaitsev E., Hoffman J. Phased array flatness effects on antenna system performance. Proceedings of the 2010 IEEE International Symposium on Phased Array Systems and Technology (ARRAY).

[16] Arnold E.J., Yan J.B., Hale R.D., Rodriguez-Morales F., Gogineni P. (2014). Identifying and compensating for phase center errors in wing-mounted phased arrays for ice sheet sounding. IEEE Trans. Antennas Propag..

[17] Famoriji O.J., Shongwe T. (2024). Modeling of coupled structural electromagnetic statistical concept for examining performance sensitivity of antenna array to distortion at millimeter-wave. Appl. Sci..

[18] Xu L., Zhang Z., Yang F., Chen Y., Hu J., Yang S. (2025). A dual-polarized leading edge wing conformal tightly coupled dipole array with enhanced low-scattering characteristics. IEEE Trans. Antennas Propag..

[19] Lian S., Wang W., Lou S., Li P., Xu W., Zhang W. (2025). Analysis of scattering characteristics of the cylindrical conformal arrays with variable curvature based on infinitesimal dipole model. IEEE Antennas Wirel. Propag. Lett..

[20] Zhang Z., Yang F., Qu S., Chen Y., Hu J., Yang S. (2021). In-band scattering and radiation tradeoff of broadband phased arrays based on scattering-matrix approach. IEEE Trans. Antennas Propag..

[21] Li P., Qu S., Yang S., Hu J. (2023). Fundamental relation between phased array radiation and scattering fields. IEEE Trans. Microw. Theory Tech..

[22] Wang Y., Yan B., Yu R., Fan M., Duan Z., Ma Z., Fu H., Wang Z., Yu K., Wu W. (2023). Sub-array level structural compensation method for radiating and scattering performance of array antennas. IET Microw. Antennas Propag..

[23] Zheng T., Xiang B., Lin S., Wang W., Lian P., Cui H. Active compensation of reflector antenna structural deformation based on deep BP-neural network. Proceedings of the 2024 IEEE 7th International Conference on Electronic Information and Communication Technology (ICEICT).

[24] Wang S., Wang J., Xu Z., Wang J., Li R., Dai J. (2024). Deformation intelligent prediction of titanium alloy plate forming based on BP neural network and sparrow search algorithm. J. Mar. Sci. Eng..

[25] Zhang S., Liu G., Cheng C. (2024). Application of modified BP neural network in identification of unmanned surface vehicle dynamics. J. Mar. Sci. Eng..

